# Exploring Managers’ Perspectives on MNCH Program in Pakistan: A Qualitative Study

**DOI:** 10.1371/journal.pone.0146665

**Published:** 2016-01-12

**Authors:** Mariyam Sarfraz, Saima Hamid

**Affiliations:** Health Services Academy, Islamabad, Pakistan; University of South Australia, AUSTRALIA

## Abstract

**Background:**

Pakistan’s Maternal, Newborn and Child Health (MNCH) Program is faced with multiple challenges in service delivery, financial and logistic management, training and deployment of human resources, and integration within the existing health system. There is a lack of evidence on managerial aspects of the MNCH program management and implementation.

**Methods and Findings:**

This study used qualitative methods to explore the challenges national, provincial and district program managers have faced in implementing a community midwifery program in province of Punjab while also exploring future directions for the program under a devolved health system. While the program had been designed in earnest, the planning lacked critical elements of involving relevant stakeholders in design and implementation, socio-demographic context and capacity of the existing health system. Financial limitations, weak leadership and lack of a political commitment to the problem of maternal health have also had an impact on program implementation.

**Conclusions:**

Our study results suggest that there is a need to re-structure the program while ensuring sustainability and collaboration within the health sector to increase uptake of skilled birth attendance and improve maternal health care in Pakistan.

## Introduction

Since the introduction of the primary health care (PHC) concept (Health for All, Alma Ata), community based health worker (CBHW) programs flourished substantially, especially in developing countries [1–3]. These programs have served as a panacea for the human resource for health shortages [4] while delivering PHC services at scale. These community based health workers, usually women, provide home based care for high priority health areas such as maternal and child health and nutrition, access to family planning services, and control of diseases such as malaria, tuberculosis, polio, measles and HIV/AIDS [5]. The potential of community based health workers is especially useful in increasing access, reducing inequality and improving key health indicators in rural and underserved populations [1]. A change in economic policies and political ideology, World Bank’s ‘Health Sector Reforms’ economized health and replaced the PHC in the 1990s [6]. The PHC concept has, however, had a renaissance in the past decade [1–3], with a renewed interest in scaling up the programs for increased services delivery coverage through effective management, while remaining within ambits of the existing health system.

Pakistan’s first community health worker program, National Program for Family Planning and Primary Health Care (NPFPPHC), commonly known as the Lady Health Worker (LHW) Program was initiated in 1994 [7]. This program was a major initiative, based on the International Conference on Population and Development’s Program of Action, for providing universal health coverage. The program currently employs over a 100,000 female, lay health workers for delivering 22 core tasks, covers almost all districts in Pakistan with the LHWs recognized as an integral part of the primary health care system [7]. Although the scale up has been phasic, management of the program has been turbulent due to frequent turn over of management and logistics staff, inefficient disbursement of funds and supplies and a limited integration with the health system and other health related programs [8].

In 2006, Pakistan initiated another community health worker based program, titled Maternal, Newborn and Child Health (MNCH) program with a bid to improve maternal health indicators, particularly those related to the MDGs. Under this program, midwives were trained and licensed to practice maternal health care services (Ante Natal Care, Delivery, Post Natal Care) in rural communities, with the aim to increase skilled birth attendance and reduce maternal mortality [9]. So far, research body on this program reports that this program is faced with implementation challenges in service delivery, financial and logistic management, training and deployment of human resources, and integration with the health system [9–14].

The government systems in Pakistan underwent a major restructuring under the democratic reform of devolution in 2011 resulting in a redefinition of the health related mandates at federal, provincial and district levels [15–17]. Theoretically, devolution provides enhanced opportunities for provinces to introduce reforms and have prioritized provincial health policies. However, in case of Pakistan, this reform is a daunting challenge due primarily to lack of a national health policy and provincial health strategies [15, 18, 19]. Under this reform, all functions of the federal ministry of health, except the vertical programs’ management have been devolved to the provincial health departments [15]. The vertical programs will continue to be managed by the Planning Commission till the year 2014 [20], following which plans are underway to integrate the LHW, MNCH, Nutrition and Immunization program under one umbrella [21]. Considering the weak provincial policy units, unstable district health systems, inadequate health sector financing, and lack of quality assurance standards for health, management of the HR intensive community health worker programs pose a daunting challenge for the provincial health departments.

The existing literature on Pakistan’s community midwifery program focuses primarily on service delivery, distribution and access aspects of the deployed midwives. There is a lack of evidence on managerial aspects of the MNCH program management and implementation. The present paper’s objective is to explore the challenges national, provincial and district program managers have faced in implementing a community midwifery program in a rural district of Punjab and the future directions for the program under a devolved health system.

## Methodology

The data presented in this paper are part of a larger mixed-methods study that aimed to assess challenges faced by the Pakistani CMWs in providing services to women in rural Punjab, Pakistan. Data for this study were collected over a 3-month period in 2011 in district Attock, Punjab. To explore the range of challenges involved, qualitative data were collected from program managers, community midwives and local community members (mothers, their husbands and mothers-in-law). Alongside, a comprehensive review of relevant policy and planning documents was also conducted.

### Study setting

District Attock was selected for this research as it was among the first regions in which MNCH program was launched and had sufficient numbers of CMWs deployed, for a significant time period (almost 3 years), to assess coverage. The overall development, geographical and social characteristics of the district matches that of Northern Punjab and Khyber Pakhtunkhwa provinces of Pakistan. Furthermore, MNCH program in District Attock is funded and managed by the Government of Pakistan with no donor support. The program hence has resource constraints reflective of the situation in most of the districts of Pakistan. This unique perspective of the district made this area an ideal location for exploring the challenges faced by community based health workers in providing maternal health care services.

### Data collection procedure

Data for this manuscript has been drawn from program policy documents reviewed and interviews conducted with program managers. The reviewed policy documents included Planning Commission documentation (PC– 1) and Deployment guidelines for Community Midwives. These policy documents detailed program objectives, interventions to be adapted and services to be provided to improve infrastructural and behavioral issues associated with low utilization of maternal health services in rural areas of Pakistan. Deployment guidelines developed for community midwives were also reviewed in detail to develop an understanding of CMWs’ scope of work and responsibilities.

Open ended, in-depth interviews were conducted with purposefully selected key actors involved in program planning and implementation process at the federal, provincial and district level, namely nine national, provincial and district program managers. Group interviews were conducted with seven (07) participants (Table 1).

**Table 1:** Interview respondent characteristics.

S. No	Respondent	Placement/ Location	Association with program (years)	Role in program
1	National Program Manager	Federal	1	National program implementation, financial management and sustainable integration
2	Focal Person–Development Partner	Federal	6	Midwifery Program design, development, implementation and financing strategy
3*^1^	Registrar–Pakistan Nursing Council	Federal	6	Regulation of nursing and midwifery education and practice
4*^1^	Midwifery Registrar–Pakistan Nursing Council	Federal	4	Regulation of midwifery education and practice
5*^2^	Provincial Program Manager	Provincial office	5	Provincial program management and scale up
6*^2^	Deputy Program Manager–CMW Component	Provincial office	5	Management of the CMW component–training, deployment, monitoring
7*^3^	EDO Health—Attock	District Office	2	Financial and Administrative management
8*^3^	Public Health Specialist—Attock	District Office	4	District level program implementation and integration
9*^3^	Social Organizer—Attock	District Office	4	CMW supervision and monitoring

* Indicates group interviews

All the interviews were conducted in interviewees’ offices at a time convenient to them. Interview guides were used to obtain information from the interviewees. Interview questions developed and refined by the core research team were aimed at soliciting experiences and opinions of the participants about the study question.

### Data analysis

All interviews were recorded and transcribed into English for further analysis. A random sample of the transcripts was checked by the authors for translation accuracy.

A thematic content analysis approach was adopted for analyzing content of the interviews, as an on-going iterative process through which issues and challenges regarding program management and implementation were identified early on. This allowed for a rigorous probing in subsequent interviews [22]. Open codes were developed and process was repeated for all transcripts. Emergent categories were identified without imposing a priori categories [23, 24]. Based on the identified categories, an initial thematic framework was developed, discussed iteratively between the authors and adjusted where appropriate. The identified themes, sub themes and categories conformed to the Health Policy Analysis triangle defined by Walt and Gilson [22], which was then used as a framework for explaining the findings.

### Trustworthiness

The study observed various procedures to enhance the credibility of the research findings. All the policy makers involved in the implementation of the program were included in the study. Data triangulation included use of policy documents and primary data collection through individual and group interviews. A peer coded two interviews independently and codes were reviewed for consistency. Throughout the data collection and analysis debriefing within research team (which comprised of public health professionals with experience in conducting qualitative studies), peer discussions and respondent validation were undertaken, thereby ensuring interpretative accuracy of the results.

### Ethical clearance

Ethical clearance for this study was obtained from bioethics committee of Pakistan Medical Research Council. A formal letter was sent to each interviewee to explain objectives of the study while requesting an appointment for conducting the interview. Interviewees were assured anonymity. Written, informed consent was obtained from all participants prior to the interviews, while ensuring complete anonymity and confidentiality.

## Results

In depth interviews with nine (09) main actors in Pakistan’s MNCH program, namely the national, provincial and district managers, development partner and representative of the midwifery training regulatory authority (Pakistan Nursing Council–PNC) were conducted. The interviews were analyzed using the policy analysis framework developed by Walt and Gilson [22]. Results of the analysis are detailed under four themes identified using the framework.

### Theme I: Relevant stakeholders not involved in program development and implementation

Stakeholders are at the heart of policy and program development, influencing program design, content, strategies, and implementation modalities; however, they cannot be separated from the organizations within which they work. This theme attempts to identify the different actors involved in the MNCH program development process, the level of influence they exercised and their authority within the context of this program.

#### Program planning and policy development

The Maternal, Newborn and Child Health program for Pakistan was developed in the wake of an MNCH strategy, launched in 2005. The main actors involved in the development of the MNCH PC -1 were the then Ministry of Health (MoH) and Department for International Development (Dfid).

The respondents shared that during development of the program policy and implementation plans, relevant personnel were not involved. Although the Director General for Health was part of the team developing the program but inputs were not taken from provincial and district Health Departments. Principals of Nursing Schools where midwives were to be trained were also not engaged in this process; especially planning pertaining to training and deployment of midwives for community services.

‘DG Health was involved in planning, but not the EDOs and Principals of Nursing Schools. We have Nursing instructors with facility-based training skills, but we do not have a single trained midwifery tutor in Punjab to teach midwifery skills for community bases services.’ (MNCH Manager)

Community based Midwives, a major component of MNCH program were envisaged to provide home based maternity care in rural areas. This was similar to the flagship program titled National Program for Primary Health Care and Family Planning (NPPHCFP) implemented for providing primary health care and family planning services through lay community health workers (Lady Health Workers–LHW) initiated in 1994; however, the national manager for this program was also not involved in planning and development of the MNCH program.

#### Selective stakeholder commitment

MoH developed the PC-1 detailing objectives, implementation strategies, operational arrangement and estimated costs. MoH and Dfid each committed to fulfill fifty percent of the program costs; UNFPA, USAID, UNICEF and other international organizations financed selected components in the program, through MoH. UNFPA was the main sponsor for deployment of CMWs in selected districts; USAID, through its program PAIMAN sponsored supplies for CMWs in its ten adopted districts while the remaining were supported through the funds available to MNCH from MoH.

#### Political and donor interferences

All the respondents reported external influences from donors and prominent local political figures in program design and implementation. The national and provincial program managers highlighted that international partners’ agendas influenced program design, its components, and the services package to be included. Allocation of budget for a preferred program component was hence driven by the donors.

‘The partner organization and Government of Pakistan were to contribute 50% each to the total cost of the program. The partner however, did not follow through with the payments on time’. (National Manager)

Political interference was reported at all levels, affecting allocation of funds, program implementation, hiring of staff including candidates’ selection for midwifery training.

“We asked the district officials to conduct a mapping study of their districts to identify number of villages and midwives required for each district but, unfortunately, a tug-of-war started there for control of finances, and due to political interference the districts started haphazard and immediate recruitment’ (Development partner)

### Theme II: Process–centralization hindering implementation

In analyzing the program implementation process, it was noted that the important stages of the health policy development (agenda building, planning, implementation, monitoring and evaluation) were followed but not completed and program planning experienced problems that affected implementation process. Although the program was developed to cater to current maternal health indicators of Pakistan but in this process involvement of provincial and district governments in agenda building and program planning was found to be almost nonexistent.

The specific problems, which had an effect on the program planning and implementation process, were centralization, shortage of trained public health professionals (specifically midwives), influence of a narrowly focused biomedical model of health in midwifery training and financial resource constraints. Centralization also hindered a wider participation from other important stakeholders such as district health departments, NGOs, and professional groups of nurses and midwives.

#### Centralized administrative and financial management

Interviewees at provincial and district levels expressed frustration with limited financial and administrative authority delegated to them. The provincial MNCH department was dependent on quarterly release of funds from the national MNCH for managing procurements and staff salaries for all districts.

Similarly, the district MNCH officials did not have any financial authority with limited administrative powers. Managers found this limiting authority to be frustrating as they could not purchase requisite materials when needed nor did they have power to hire or layoff district program personnel. Similarly, funds transferred from national to provincial to district program offices were frequently delayed resulting in delayed transfer of staff salaries. The provincial and district management countered this problem by re-appropriating funds from other budgetary heads for managing routine affairs of the program.

Considering that devolution of the health ministry was imminent at time of data collection, there were no post devolution plans for transfer of authority, financial management and long-term sustainability. The Punjab health department was at the time developing plans for an integrated reproductive health and nutrition program. However, it has been four years post devolution and till date an integrated program is yet to materialize. It was the general opinion at district level that all vertical programs should be merged and integrated within district health department. The development partner raised concerned with the limited capacity of the provincial health departments for managing this program after devolution.

‘If after the devolution, Program is devolved to the provinces, then I don’t know how they will run it’ (Development partner)

‘After the devolution, there will only be funding flow form the federal government. There is a lot of confusion, things are not clear yet. But we understand that the federal funded programs will stay on and Punjab will have an integrated RH program’. (Program Manager)

#### Placement of program personnel

There was a considerable delay in hiring key program personal at all three administrative levels. National manager for the program was hired three years after initiation of the program and in interim period the program was run on ad-hoc basis. Punjab Provincial manager revealed that several districts still did not have a regular Public Health Specialist for managing the program at district level and this responsibility was given as an additional charge to either a district health official or the national program’s district official. Most of the district officials working for the program were on deputation from the health department. This led to the issue of frequent staff transfers thereby affecting implementation of program activities in the districts.

#### Registration and deployment of midwives

Regarding training and management of community based midwives, all respondents highlighted registration and deployment, key component of the MNCH program, as a particularly weak area requiring much improvement for achieving intended targets.

A common concern raised by provincial and district respondents was that of registration of the trained midwives prior to their placement in rural communities. At the time of data collection, 4700 midwives had been trained but only 2800 had been registered and issued license to practice. The delays in registration were primarily due to inability of graduates to pay registration fees and incomplete documentation sent by the MNCH program to PNC. The Registrar PNC informed that registration fee issue was resolved through donor funds. However, issue of missing requisite documents was still causing delays in registration.

Respondents at National program management level highlighted that midwifery component of the program took three to four years to establish with the result that although midwives were trained but due to delays in registration and absence of any deployment guidelines, several trained midwives had either gotten married and relocated to some other locality or taken up a private sector job. This resulted in considerable loss for the program given the considerable costs involved in training CMWs.

‘The program needs a strategy to keep the CMWs attached to the program as it will be difficult to bring back those lost to the private sector’. (Development partner)

#### Fragmented monitoring and evaluation process

A monitoring and evaluation process for assessing progress of the program had been defined and devised but not implemented. Delivery of supplies to strengthen health system for providing maternal and childcare was recorded but this information was not associated with service delivery targets. Similarly, the program had plans to integrate with national Health Management and Information System (HMIS) but it had not been linked up with it. Although the district officials continued to send a monthly report to provincial head quarters, this information was not reflected in HMIS.

Reference to field supervision and monitoring of midwives, the program had divided it into technical and administrative components; technical field monitoring of CMWs’ performance was responsibility of the district tutors and administrative supervision was domain of a Lady Health Supervisor (LHS), an employee of the National Program. Issues highlighted by district program officials were that this fragmented mode of monitoring and supervision was ineffective and not comprehensive. Neither of the two intended field supervisors had been given a formal training or a job description of their task. The tutors at times were not available for making monthly visits due to their routine job activities. The LHS, on the other hand, was not given a salary or other support for field visits and hence considered such supervisory visits additional burden.

The CMWs were trained to refer complicated and emergency cases to a health facility providing obstetric care. They were given a referral form for such situations; however, the referral sites had not been defined and no mechanism had been defined to solicit feedback from the facilities regarding referred cases. These lacunae in program design made it difficult to monitor progress and performance of a CMW and subsequently make requisite improvements.

### Theme III: Deficient program content

In analyzing the content of MNCH program, an analysis of the program documents and deployment guidelines developed for CMWs was undertaken. PC-1 of MNCH program defined the objectives of the program which were geared to increase skilled birth attendance for achieving MDGs 4 and 5. The CMW were proposed to be a cadre of community-based health workers, who would meet the international definition of skilled birth attendants. However, in practice, there are several shortcomings in the implementation, as explained in the previous section.

The training curriculum for CMWs was adapted from international sources, based on a biomedical model and was hence not context specific. This was compounded by a dearth of CMW tutors across the country thus affecting teaching, which was done primarily by nursing tutors and obstetricians in a clinical setting. Service structure and career path for the inducted CMWs was also not defined. The program intended to increase SBA but development of training institutes for CMWs had not been addressed. The PC-1 had not defined mechanisms for the financial sustainability of the program beyond 2012.

#### Deficiencies in midwifery training

Training curricula of midwives falls under the domain of Pakistan Nursing Council (PNC), the regulatory body for nursing and midwifery practice in Pakistan. PNC revisits and revises nursing and midwifery curricula every three years based on recommendations of teaching faculties; however, the midwifery curricula had not been revised prior to initiation of midwifery trainings under MNCH program. The PNC respondent highlighted that irrelevant teaching faculty was training the midwives and their curriculum had more of theoretical content rather than practical and field based training. Also teaching program was not standardized across the country resulting in varied level of training conducted within the resources available to different midwifery schools. In schools where medical doctors taught, training was deficient in terms of field practice. Similarly, the midwifery curriculum did not specify practices that a community midwife should adopt and avoid in a community setting.

‘Most of the trainers have no concept regarding the role of CMWs so they teach them biomedical models of reproductive health, genetic abnormalities, sexology. A midwife does not need that. They should have trainers from the same field (nursing) so that they develop the concept of theoretical, clinical and practical training required for CMWs’. (PNC representative)

Practical training of midwifery students is conducted at the District Headquarter Hospital (DHQ) where nurses also get trained. The current practice did not provide them with the requisite time for learning and practicing maternal and delivery care skills. Furthermore, the lack of trainers was also highlighted by the district program officials.

‘The DHQ is small with a lot of students attached i.e. nurses, LHVs and now CMWs. Number of trainees in DHQ Attock is more than the trainers can cover (more than 200 students in each class). Nurses and LHVs dominate the training so the CMW can only observe (in the DHQ)’. (Public Health Specialist)

All program managers were concerned about the high attrition and drop out of trained midwives and midwifery students as this was a waste of program’s limited funds. One of the factors leading to drop out during training was lack of appropriate facilities in hostel accommodation and arrangements for married students and their children.

‘There are funding provision in PC1 for civil work, but surprisingly there is nothing for support staff (sweepers, cooks, housekeepers) or utility bills. In most places students are cooking themselves. Should they concentrate on training or cooking?’ (Program Manager)

Another factor highlighted as leading to drop out was lack of proper orientation regarding job description, large catchment population, lack of transport for field visits, a low pay of PKR 2000 (approx. USD 20) and lack of a career path.

‘We do not give them proper orientation (information) either, before start of training. We should inform the families and candidates about the scope of work before hiring so that only those should join who can willingly work in community setting’ (Program Manager)

#### Program sustainability not ensured

Other concerns raised by national and provincial program managers were lack of a retention policy for the trained midwives and long-term financial sustainability of the program.

The trained midwives were required to serve under MNCH program for a period of three years (mandatory service bond) during which time they were given supplies and a retaining fee. After this time period, the program had no planned policy for long term follow up and options for retention in program.

Similarly, with the devolution imminent, the interviewees reported that there were no final plans for program continuity. The PC– 1 for all vertical programs were planned till the year 2014 during which time they would receive an annual budget from the federal budget. However, after that time, provinces would be responsible for managing all aspects of the programs including procurement, human resources management, field operations, etc.

‘Long-term sustainability of the program is shaky as government has no money’ (Development partner)

Provincial and district program managers identified a general lack of ownership by the health department as a major factor, which could impact long-term sustainability of the program. They were of the view that in the current scenario of donor funds, the program was continuing its operations within the resources made available to them but there should be more focus on results based financing

‘There is no clarity on the future modalities of Program management and directions / guidelines form the federal level. The structural integration as proposed in Punjab PC1 is also unclear.’ (MNCH Manager)

### Theme IV: Contextual influences on the program

The MNCH program had two main components, the first of which was to strengthen district health department through technical and managerial capacity improvements, streamline services for provision of basic and comprehensive emergency obstetric and newborn care (EmONC) and integration of MNCH services at district level. The second major component of the program was to launch a cadre of community based skilled birth attendants [25].

#### Limited provincial health department capacity

In analyzing program context, it was revealed that political instability influenced all levels of program leading to weak institutions, low priority for health services provision and a delayed program implementation. The prevalent financial crunch impacted overall governmental expenditures on health with the result that release of funds to provincial MNCH program was delayed. Despite provision of requisite inputs (equipment, essential medicines, skills enhancement of available HR and finances) for provision of EmONC service at primary health care level (at Rural Health Centers–RHC), district health system was unable to follow through. However, systemic public sector health department problems of vacant positions, absenteeism and deficient infrastructure compromised service delivery. All these factors hindered effective provision of quality services.

Respondents at national level flagged the concern of deficient service delivery in districts and their compromised capacity to deliver services at par with those proposed in the program PC-1. They further admitted that they were forced to work with whatever resources were available to them.

‘By now, we should have spent almost 55–60% of the allocated funds, as opposed to 30% that we have received so far. Overall, in this Program we have not moved as it was supposed to’. (Development partner)

#### Indigenous context not incorporated in midwifery component

The community based skilled birth attendant (CMW) component of the program was not entirely context specific. The defined selection criteria (as per PNC regulations) required a candidate to be between the ages of 18 to 45 years, preferably married, have ten years of formal education with 45% score in secondary school exam. These selection criteria were compromised in several districts. Young married women were not permitted by their family to join the program and the older married women had compromised knowledge. In many cases institutes had to teach the midwifery students basic reading and writing skills as well. Students were required to reside in hostel accommodation during training period but because of lack of facilities, lack of accommodation for married women, many of the students dropped out of the program. The students who remained in training were mostly unmarried young girls. Furthermore, there was no policy in the program pertaining to maternity leave and benefits for midwifery trainees.

‘Most of the students are from poor families, and have no support system to leave their children at home while attending classes. According to PC-1, this (midwifery) has to be residential training; now what would a married woman with children do? We have given relaxation in Punjab; come in the morning and leave at 3:00 p.m. but patient load is more in evening or at night. So their training suffers. Tutors complain that married students are frequently absent, sometimes their kids are not well, sometimes, mother-in-law is sick, etc. etc. The young unmarried students are so young that in our culture it is not acceptable that they talk about reproductive health. To expect women to accept them and have them conduct a delivery is a big challenge. These are strange problems; unless you experience these, you don’t foresee them and hence don’t plan to address them’. (Provincial Manager)

The midwifery regulatory authority highlighted that they have been requested to revise selection criteria so that single women and those with lower grades may also apply thereby increasing the pool of applicants. Registrar PNC highlighted that some of the districts had relaxed the selection criteria at their own behest. This had caused problems for graduates in setting up a practice in community settings.

“Even if a woman is 40 years of age but is not married, omen do not want to avail maternity and delivery services from them as they have a pre-conceived notion that they are not aware of maternal care issues.

(Larki agar 40 saal ki bhi ho laikin shadi shuddah na ho to loag phir bhi us say delivery nahi kerwatay kyun keh un kay khiyal mein us ko in baton ka kia pata)” (PNC representative)

Local cultural values had also not been incorporated in deployment guidelines whereby each CMW is allocated a catchment population of 10000, with no transport facility to move about in designated area. The patriarchal culture and religious values practiced commonly in Pakistani society, especially rural areas, have particularly hindered mobility of young CMWs in their catchment areas.

Existence of private maternity care providers, which include both trained and untrained professionals, has also not been factored in design of the CMW program. Preference for private care providers [10, 26] has made it difficult for young, single midwives to establish practice.

## Discussion

The study results provide valuable insights from program managers involved in development and implementation of a community health worker based, vertical program for maternal health care. The program failed to achieve its desired impact primarily by not involving key stakeholders from within the health system, midwifery training institutes and parallel program in the design stage. This resulted in implementation struggles at all levels. The MNCH program had been designed for a centralized health system with the result that following devolution, the program is struggling to regain its already weak footings. The main reason for this appears to be a lack of health policy makers and planners ‘capacity for managing health services in a devolved system. Furthermore, financial crisis and lack of a sustainability plans appear to have affected optimum program functioning.

### Uninterrupted financial support

Financial sustainability of programs with pre-defined targets needs an uninterrupted support to enable the program to achieve its goals. Major reasons for low and interrupted financial support of the program appear to be lack of funds, devolution of the health services and the financial crisis in health sector. A lack of funds and delayed disbursement has been cited as the major reason for the MNCH program not being able to establish and function optimally. A chronological dissection of the program management reveals breaches in financial flows by all the development partners, namely government and international development partners. The program also lacked a sustainability plan; it was a planning presumption that CMWs would be assimilated within the existing health infrastructure and be accepted by the community without any resistance from other health care providers. However, considering this as a handicap due to the program being in ‘infancy’ stage, the program had started functioning as per the program plan, albeit with deficits. The successes achieved by the program in terms of streamlining financial management received a major setback due to the devolution of health services management to the provinces. At time of devolution, financing mechanisms for the provinces were yet to be developed, with the result that financial disbursements for the programs have been retained at the federal level till the year 2016. This has added a further complexity; implementation and administrative management at provincial health department with financial control at federal level is still a hurdle for achievement of program targets.

Historically, health sector in Pakistan has been allocated a budget of less than 1% annually; primarily due to focus on defense and development. Lack of a political commitment for social sectors, natural and manmade disasters and a global financial crisis have been cited as the common reasons for a low budgetary allocation for the health sector.

### Devolution of financial and administrative powers to district health units

The government of Pakistan has been ‘experimenting’ with administrative reforms of decentralization and devolution since the mid 1990s [27]. Prior to the 18^th^ constitutional amendment, health institutions were decentralized to provincial level and made Autonomous Medical Institutes in 1990 under the World Bank funded Social Action Program [27]. The aim was to develop a devolved management structure for the health sector at District level. However, this did not have the favorable results, as powers were not delegated to the district officials in financial and administrative decision-making, with the result that the project was closed, unfinished in 1998. This was followed by the Punjab Medical and Health Institutions Ordinance 1998, under which, medical colleges and allied hospitals were made autonomous. This reform again was not implemented to take full effect due to weak and haphazard documentation; the project was rolled back with the change of government [27, 28]. The latest reform, in effect and unlikely to be rolled back is the 18^th^ Constitutional Amendment, which has effectively devolved the health services to the provinces in its entirety [20]. Despite the repetitive bureaucratic experimentation with autonomy, decentralization and devolution, the health sector is still struggling to understand the merits of decentralization and implement it in its true spirit.

This confused state of affairs is apparent at the MNCH program management level as well, with national, provincial and district managers struggling for effective implementation but not unable to achieve due to lack of administrative and financial controls. Considering the extent of MNCH Program services and the nature of its functions, it is imperative that financial as well as administrative authority for program management be entrusted to the district health department. This would give the program an operational fiscal and administrative space for improving service delivery within the district. Dependence on a centralized system for management will continue to have an impact on overall efficiency of the program.

### Clearly defined collaboration

The MNCH program, in theory, relies heavily on collaboration with the National Program and the public sector health system at district level. However, there is a need to clearly define the parameters for this collaboration, identifying the roles and responsibilities for each of the partners and their stakes. Lack of clearly assigned roles is resulting in the program not functioning at its optimum. The CMWs, an instrument of the program for achieving an increase in uptake of skilled birth attendance, are faced with multiple setbacks in establishing their services due to a lack of support from the district health system, fragmented supervision and vague feedback [9]. Program management at federal and provincial levels need to develop comprehensive terms of references for the input required from the partners and guarantee that financial and administrative investments are made to ensure achievement of the defined relationships.

There is also a need to define clearly the ambit of the program, taking care to avoid duplication of interventions already in place for improving maternal health and increasing uptake of skilled birth attendance. At present, two other public sector organizations/programs are concurrently providing maternal care services–the NPFPPHC and the district health system. The LHWs are required to provide ANC care and refer pregnant women to the local Rural Health Center (RHC), where women are given an incentive for a facility based delivery along with free delivery services [9], under the MNCH program’s initiative of 24–7 maternal care services [29]. In this existing scenario, it is unlikely that the midwifery component of the MNCH program will be able to establish itself in the rural communities.

### Continued and committed leadership

The MNCH program has been subject to insufficient (national leadership). The national manager for the program was appointed three years after the program plan had been developed and implemented; in the interim period, the then Ministry of Health officials managed program as an additional charge. However, the national manager worked with the program for a limited time period of two years due to the changes in health sector following the 18^th^ Constitutional Amendment. The program was then handed over to provincial managers for regional management. This lack of a committed leadership resulted in the program facing challenges in implementation including financial lapses, provincial implementation, deployment of personnel, partnerships and coordination within and between the other government run programs and health system. Success in achieving the targets of vertical programs is as much dependent on the program planning and implementation as it is on the management and leadership [30]. The other national program operating through community based health factors has been able to improve the identified health indicators primarily due to existence of continued management structure and leadership [31, 32]. Considering that the program is now devolved to the provincial level, committed leadership and strong management will continue to be a driving factor for improving the implantation of the program and successful integration within the existing health system for long term sustainability.

## Conclusion

The administrative instability resulting from absence of a national program manager and health sector reform of devolution has had an impact on effective program implementation, delayed deployment of service providers and lack of integration within the existing health system. Health policy makers and program managers should continue strengthening the services delivery under this program by ensuring availability of trained midwives, improved quality of services, and adaptation to the contextual factor arising from the health sector devolution using context specific interventions. Renewed focus on ensuring sustainability and collaboration within the health sector is needed to increase uptake of skilled birth attendance and improve maternal health care in Pakistan.
